# Recovery of rare earth elements from low-grade coal fly ash using a recyclable protein biosorbent

**DOI:** 10.3389/fbioe.2024.1385845

**Published:** 2024-05-16

**Authors:** Zohaib Hussain, Divya Dwivedi, Inchan Kwon

**Affiliations:** School of Materials Science and Engineering, Gwangju Institute of Science and Technology (GIST), Gwangju, Republic of Korea

**Keywords:** rare earth elements, biosorption, biomaterials, elastin-like polypeptide, liquid–liquid phase separation, intrinsically disordered protein, coacervation

## Abstract

Rare earth elements (REEs), including those in the lanthanide series, are crucial components essential for clean energy transitions, but they originate from geographically limited regions. Exploiting new and diverse supply sources is vital to facilitating a clean energy future. Hence, we explored the recovery of REEs from coal fly ash (FA), a complex, low-grade industrial feedstock that is currently underutilized (leachate concentrations of REEs in FA are < 0.003 mol%). Herein, we demonstrated the thermo-responsive genetically encoded REE-selective elastin-like polypeptides (RELPs) as a recyclable bioengineered protein adsorbent for the selective retrieval of REEs from coal fly ash over multiple cycles. The results showed that RELPs could be efficiently separated using temperature cycling and reused with high stability, as they retained ∼95% of their initial REE binding capacity even after four cycles. Moreover, RELPs selectively recovered high-purity REEs from the simulated solution containing one representative REE in the range of 0.0001–0.005 mol%, resulting in up to a 100,000-fold increase in REE purity. This study offers a sustainable approach to diversifying REE supplies by recovering REEs from low-grade coal fly ash in industrial wastes and provides a scientific basis for the extraction of high-purity REEs for industrial purposes.

## 1 Introduction

Rare earth elements (REEs) are vital for modern technology and clean energy. However, their supply is concentrated in a few countries, posing challenges for the transition to clean energy. Diversifying REE sources through sustainable methods and recycling is crucial (The White House, 2022; Carrera et al., 2023). Non-traditional REE resources, such as coal byproducts, are abundant and can diversify the REE supply chain (Park et al., 2017). However, conventional REE extraction methods are technically challenging, intricate, and costly due to the low REE content and the high concentrations of competing metals found in these feedstocks. Furthermore, these REE extraction processes, especially hydrometallurgy through solvent extraction, require significant energy input and impose substantial environmental burdens (Park et al., 2017; Mattocks J et al., 2020). Hence, developing alternative technologies that enable the selective, efficient, and environmentally friendly recovery of REEs from non-traditional feedstocks is paramount to tackling their supply challenges.

Biosorption represents a potentially cost-effective and eco-friendly approach for metal recovery. Biosorption methods using natural and engineered whole cells and natural and engineered proteins have been explored for REE extraction. However, they have limitations, such as poor selectivity and operational issues. Industry and associated research fields generally ignore proteins for the REE life cycle and favor small, artificial chelators instead. However, the recent discovery of lanmodulin (LanM), a natural chelator, offers a sustainable alternative to conventional extraction methods with exceptional selectivity, especially for light REEs (Cotruvo et al., 2018; Deblonde et al., 2020; Dong et al., 2021; Featherston et al., 2021; Hussain et al., 2022), and has served as a technological platform for f-element detection, recovery, and separation (Deblonde et al., 2020). However, using only proteins for REE adsorption is not suitable and economical due to the short life, difficult reuse, and expensive single-use of proteins, and the extraction of desorbed REEs from free protein-based biosorbents is not trivial (Hussain et al., 2022). Harnessing LanM for the effective and efficient recovery of REEs requires biosorption material with desirable functionality, stability, and reusability. Thus, to further improve the method efficiency by enabling facile protein reuse, recent studies highlight using a solid–liquid extraction process for the selective recovery of REEs. For example, LanM was immobilized onto agarose microbeads based on thiol–maleimide click chemistry. The resulting biosorbent was used for grouped REE extraction from low-grade feedstock fly ash (FA) leachate in a flow-through format (Dong et al., 2021). In another study inspired by LanM, chimeric protein DLanM containing two copies of LanM was immobilized onto agarose microbeads using a Cnbr-activated amine condensation reaction. The fixed bed column was then packed with DLanM–agarose for the selective recovery of REEs under flow-through conditions (Cui et al., 2023). In a recent study, SpyTag–SpyCatcher (Spy) chemistry was harnessed for bioconjugation to obtain REE-binding biomaterials; SpyCatcher-fused LanM was immobilized on the surface of SpyTag-functionalized magnetic nanoparticles. The engineered biomaterial selectively adsorbed REEs from the geothermal brine and low-grade leachate of coal FA. However, these methods have associated drawbacks of a time-consuming and complex material synthesis and immobilization process, reduced protein functionality, and REE adsorption kinetics upon immobilization (Dong et al., 2021; Xie et al., 2022; Ye et al., 2023a).

The reduced protein REE adsorption activity is observed because of the low protein-loading capacity and protein denaturation by chemical reagents and materials upon immobilization. Compared to the free LanM protein, upon immobilization using thiol–maleimide click chemistry, LanM retained ∼67% of the adsorption activity, and one of its REE-binding sites was also destabilized (Dong et al., 2021). In the case of SpyTag–SpyCatcher chemistry, LanM maintained ∼80% of adsorption activity (Ye et al., 2023a). The use of toxic chemicals and reagents or stringent conditions reduces the stability/activity of proteins. Moreover, the orientation and conformation of proteins are also hard to control, which may negatively impact protein stability/activity (Ye et al., 2023b). It is also important to consider the use of a non-adsorptive support matrix that allows the selectivity of the REE binding protein to determine the purity of the recovered metal solution and avoids potential matrix-mediated sorption of non-REEs and REEs; for example, non-protein-conjugated agarose microbeads showed an adsorption capacity of 4.38 μg mL^-1^ for La^+3^ (4,380 ppb or 31 µM) at pH 3.5 (Brewer et al., 2019a; Cui et al., 2023).

We recently introduced a novel method named RExtractor, which employs a protein-based, all-aqueous approach for liquid–liquid phase separation to selectively recover total REEs. This method utilizes a REE-sensitive, genetically encoded elastin-like polypeptide (RELP) that responds to changes in temperature. The technology allows for the repeated use of RELP biosorbents, enduring multiple cycles of adsorption/desorption and phase transitions to recover Tb^3+^. Additionally, the RELP demonstrates resilience to acidic pH levels ranging from 3 to 6. The purification of RELP involves leveraging its unique phase transition behavior using inverse transition cycling (ITC), where the elastin-like polypeptide (ELP) serves as a purification tag (Hussain et al., 2022). This method is advantageous due to its cost-effectiveness and time-saving nature, eliminating the need for chromatography and not being limited by resin capacity (Meyer and Chilkoti, 1999; Hussain et al., 2022). Liquid–liquid phase separation (LLPS), also known as coacervation, is a process where macromolecular solutions undergo phase separation, resulting in the formation of a dense, polymer-rich phase and a solvent-rich phase (Dinic et al., 2021). LLPS is commonly observed in proteins containing intrinsically disordered regions, like elastin. Various factors, including temperature, salt concentration, pH, and other biomolecules, such as RNA or ATP, are studied to modulate protein LLPS behavior (Dignon et al., 2019). Elastin LLPS, for example, is driven by temperature and involves entropic interactions between hydrophobic sequences or domains and the disruption of water molecules surrounding the polymer. Inspired by this behavior, ELPs have been designed for numerous biomedical and industrial applications (Vidal Ceballos et al., 2022). ELPs exhibiting lower critical solution temperature (LCST) phase behavior dissolve in aqueous solutions below their transition temperature (Tt) but undergo phase separation into a polymer-rich, insoluble coacervate phase at temperatures above Tt (Varanko et al., 2020). This reversible LCST behavior, known as coacervation, enables the inexpensive purification of ELPs through ITC, pioneered by Meyer and Chilkoti (1999). In ITC, soluble ELPs are collected below Tt, while insoluble contaminants pellet down during cold spin, and the opposite occurs during hot spin above Tt. By adding salt (not exceeding 3 M) and heat, ELP coacervation above Tt is facilitated, leading to the formation of micron-sized aggregates enriched in ELP fusion proteins. These aggregates can be separated from other soluble cell lysate components via centrifugation (hot spin) (Hassouneh et al., 2010).

**FIGURE 1:**
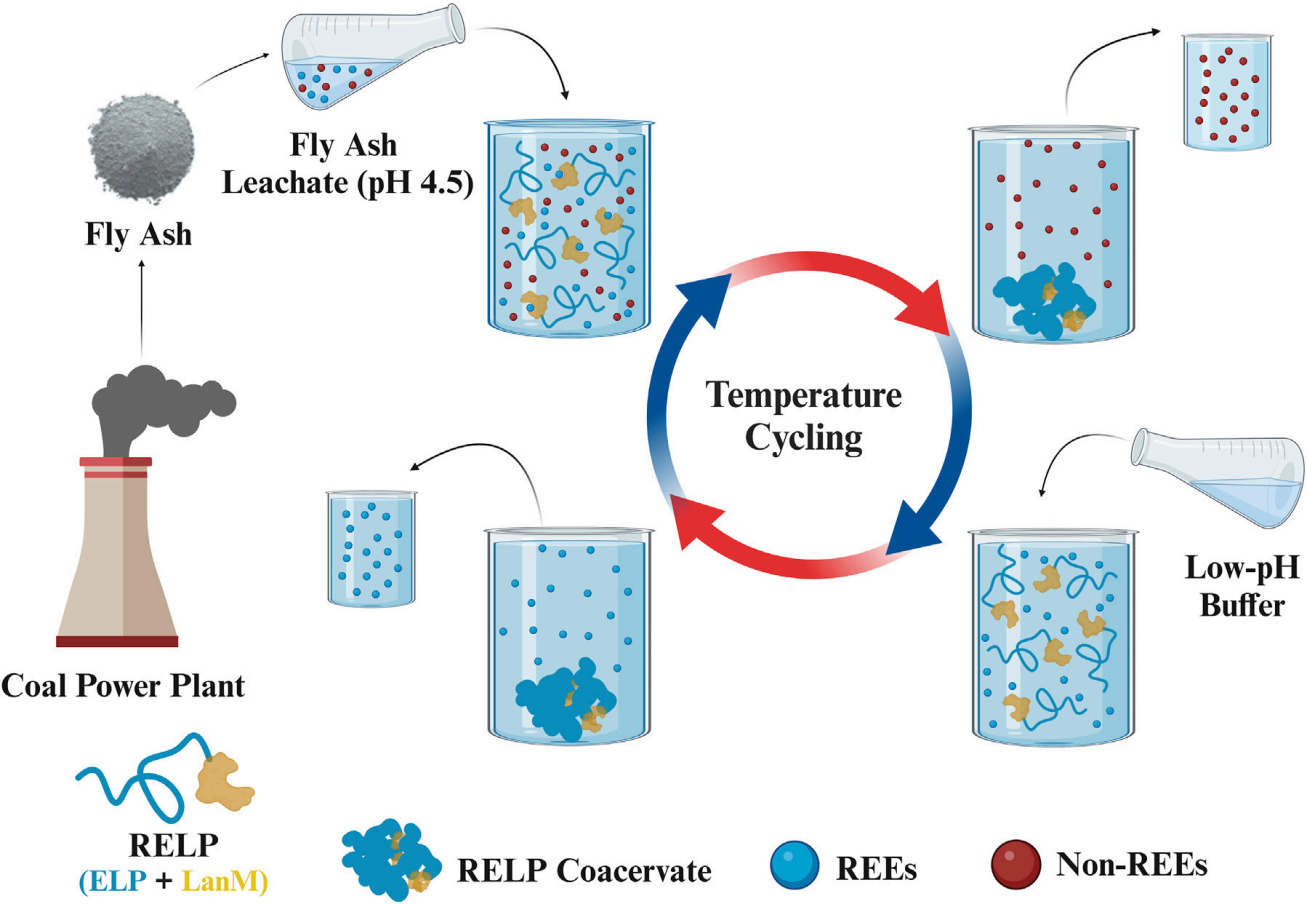
Schematic diagram (Created with BioRender.com) of temperature cycling of RELP for the selective recovery of REEs from FA leachate through the following steps: with the addition of the FA leachate to RELP solution at 4°C for adsorption at pH 4.5; phase separation above Tt and centrifugation at 37°C to remove non-REEs; solubilization and desorption of the bound REEs at 4°C in the desorption buffer; similar to step 2 to collect the coacervate and REEs. For subsequent cycles, RELP coacervates were resolubilized at 4°Q17C, with the addition of fresh FA leachate for its repeated use in the recovery of REEs.

Phase-transition efficiency of ELPs can be affected by several factors, such as ELP concentration, salt concentration, pH, and temperature. Therefore, there are two convenient ways to modulate the phase transition of ELP-fused proteins: increasing the solution temperature above the inverse T_t_ or increasing the salt concentration to depress the T_t_ below the solution temperature (Hassouneh et al., 2010). For metal desorption from biosorbents, mostly acids, salts, and ligands have been used. However, using salts in the desorption step reduced the REE recovery yield and purity (Park et al., 2016). Most of these studies focus on environmental remediation rather than extracting REEs for industrial applications, for which the purity of the recovered metals is critical. In the current study, we refrain from using salt during the desorption step by inducing the phase separation of RELPs through heating above Tt without the addition of salt (Figure 1) to enhance REE purity through temperature cycling.

The key research needed for this promising new protein-based REE recovery technology is to demonstrate its ability to recover REEs in repeated cycles from low-grade industrial or environmental samples (Hutchison et al., 2021), even at environmentally relevant concentrations. For this purpose, coal combustion residuals, including FA, have been widely investigated as a potential source of REEs (Stoy et al., 2021). Coal power plants worldwide release millions of tons of coal ash annually and are landfilled. The leaching of toxic trace elements and ash spill events make landfills a significant environmental challenge (Deng et al., 2023). The recovery of REEs from FA has several advantages, which include readily available waste products, not requiring extensive excavation, and having a fine powder nature that makes it ideal for chemical processing (Taggart et al., 2016).

The present study offers the first direct experimental evidence of a RELP for extracting REEs from unconventional, low-grade REE feedstock in batch operation and its ability to recover low-concentration REEs with high purity. This technology will enable efficient and sustainable REE recovery from FA in order to meet the REE demand while also addressing the increasing environmental concerns with coal combustion residuals. Herein, we report that the use of RELPs for the selective extraction of total REEs from FA using temperature cycling paved the way for a quantitative, sustainable design of protein-based adsorbents for REE recovery.

## 2 Materials and methods

### 2.1 Materials

Restriction enzymes, ligase, and other molecular reagents for gene cloning were obtained from NEB (Ipswich, MA, United States). The pET-24a-ELP[V150] plasmid was purchased from Addgene (ID: 67015) (Watertown, MA, United States). Macrogen, Inc. (Seoul, South Korea) synthesized all DNAs and primers used for cloning. Unless otherwise specified, chemical reagents were obtained from Sigma-Aldrich (Saint Louis, MO, United States).

### 2.2 Preparation of the coal fly ash leachate

An FA sample was collected from a coal-fired power plant operated by the Korea Midland Power Boryeong Power Generation Site Division. A measure of 5 g of FA was leached using 50 mL of 2 M HCl solution for 3 h. The leachate was centrifuged at 5,000 *g* for 15 min to remove any undissolved particles before pH adjustment using NaOH. The pH-adjusted leachate was centrifuged again to remove precipitates formed during pH adjustment. The final leachate solution obtained had a pH of 4.5. The concentrations of Y^3+^, La^3+^, Ce^3+^, Pr^3+^, Nd^3+^, Sm^3+^, Eu^3+^, Gd^3+^, Tb^3+^, Ho^3+^, Er^3+^, Tm^3+^, and Yb^3+^ were determined by ICP-MS (Agilent 7900, Agilent Technologies, Santa Clara, CA, United States). The Na^2+^, Mg^2+^, and Ca^2+^ concentrations were determined by ICP-OES (iCAP7400DUO, Thermo Scientific, Waltham, MA, United States). Samples were diluted with 2% HNO_3_ for analysis. The ICP-MS and ICP-OES results corresponded to the mean values (*n* = 3).

### 2.3 Protein expression and purification

The expression and purification of RELPs were performed as previously reported (Hussain et al., 2022). In brief, the pET-24a-RELP plasmid encoding the RELP gene was constructed by adding the LanM gene to the pET-24a-ELP[V150] plasmid. The features of the pET-24a-RELP plasmid are shown in Supplementary Material. The DNA and amino acid sequences of RELPs were previously reported (Hussain et al., 2022). After the protein expression and purification of RELP, the concentration of the purified RELP (molar extinction coefficient: 6,990 m^−1^ cm^−1^) was measured at an absorbance of 280 nm using a microplate reader (Synergy, BioTek, Winooski, VT, United States) according to Beer–Lambert’s law (Pace et al., 1995; Anthis and Clore, 2013).

### 2.4 Structure prediction using AlphaFold

The AlphaFold Colab notebook from DeepMind was used for protein structure prediction (Jumper et al., 2021), and the full RELP sequence was provided as an input. The structure was visualized using PyMOL Molecular Graphics System, version 1.2r3pre, Schrödinger, LLC.

### 2.5 Characterization of reversible phase-transition behavior

To investigate the phase transition of the RELP at different concentrations, the RELP in 20 mM MES buffer (pH 5.8) was heated from 26°C to 44°C at a rate of 1°C/2 min, and changes in absorbance at 350 nm (A_**350nm**_) were monitored using the microplate reader. The reversible phase-transition behavior over multiple cooling and heating cycles was further investigated by measuring size changes using dynamic light scattering (DLS). DLS was performed using the Zetasizer Nano ZSP (Malvern Instruments, Malvern, United Kingdom) with the 173° backscatter detector. Protein solutions were prepared in MES (final concentration of 25 μM), transferred to a DLS cuvette (precooled at 10°C), and quickly transferred into the Zetasizer. After incubation at 10°C, the first set of DLS measurements was conducted. Subsequent measurements were conducted at alternating temperatures (heating to 37°C and cooling to 10°C) for three cycles. The hydrodynamic diameter (Zavg) was calculated. Measurements were performed in triplicate.

### 2.6 REE recovery from the coal fly ash leachate

In 20 mM MES buffer (pH 5.8), the FA leachate (Table 1) and 25 µM of RELP were mixed and kept at 4°C. The coacervation above the T_**t**_ was performed using a heat block at 37°C for 10 min, followed by centrifugation at 15,000 rpm at 37°C for 10 min, and the supernatant containing unbound metals was transferred to the new tube. Next, the RELP coacervates were resolubilized in phosphate–citrate buffer (4 mM Na_2_HPO_4_ and 100 mM citric acid, pH 2.2) at 4°C for 10 min for the desorption of bound REEs from RELPs. To separate the RELP and desorbed REEs, coacervation above T_t_ was triggered as described previously. The supernatant was recovered and used to analyze the recovered metals. The obtained RELP coacervates were resolubilized in fresh coal fly ash leachate for subsequent cycles (up to four cycles). The concentrations of the recovered REEs were determined using ICP-MS. The percentage of the metal recovered was calculated relative to the amount of metal added initially.

**TABLE 1:** Initial chemical composition of the coal fly ash leachate.

Metal		Concentration (µM)
REEs	Y	1.376
	La	0.893
	Ce	1.710
	Pr	0.211
	Nd	0.829
	Sm	0.171
	Eu	0.035
	Gd	0.169
	Tb	0.025
	Dy	0.141
	Ho	0.028
	Er	0.076
	Tm	0.010
	Yb	0.060
Non-REEs	Mg	4265
	Na	128387
	Ca	19701

### 2.7 Batch experiment to determine the recovery ability of the RELP for low concentrations of REEs

The RELP was incubated in four different solutions containing varying concentrations of REE (Tb^3+^) (1 µM to nM range) and equimolar concentrations of Mg and Zn (approximately 1 mM each). The REE was recovered as described above, except for the desorption of bound metal using citrate buffer (100 mM citric acid, pH 1.8). The concentrations of the recovered metal in the filtrate were analyzed using ICP-MS.

Metal ion purity, fold increase, and yield are defined as

$${Purity}_{{Tb}^{3+}}=\frac{C_{{Tb}^{3+}}}{C_{{Tb}^{3+}}+C_{{mg}^{2+}}+C_{{zn}^{2+}}},$$

$${Fold\;increase\;in\;purity}_{{Tb}^{3+}}=\frac{{Purity}_{{Tb}^{3+}}in\;recovered\;solution}{{Purity}_{{Tb}^{3+}}in\;initial\;solution},$$

$$Yield=\frac{Recovered\;C_{{Tb}^{3+}}}{Initial\;C_{{Tb}^{3+}}\;}.$$

## 3 Results and discussion

### 3.1 Characterization of the biosorbent

**FIGURE 2:**
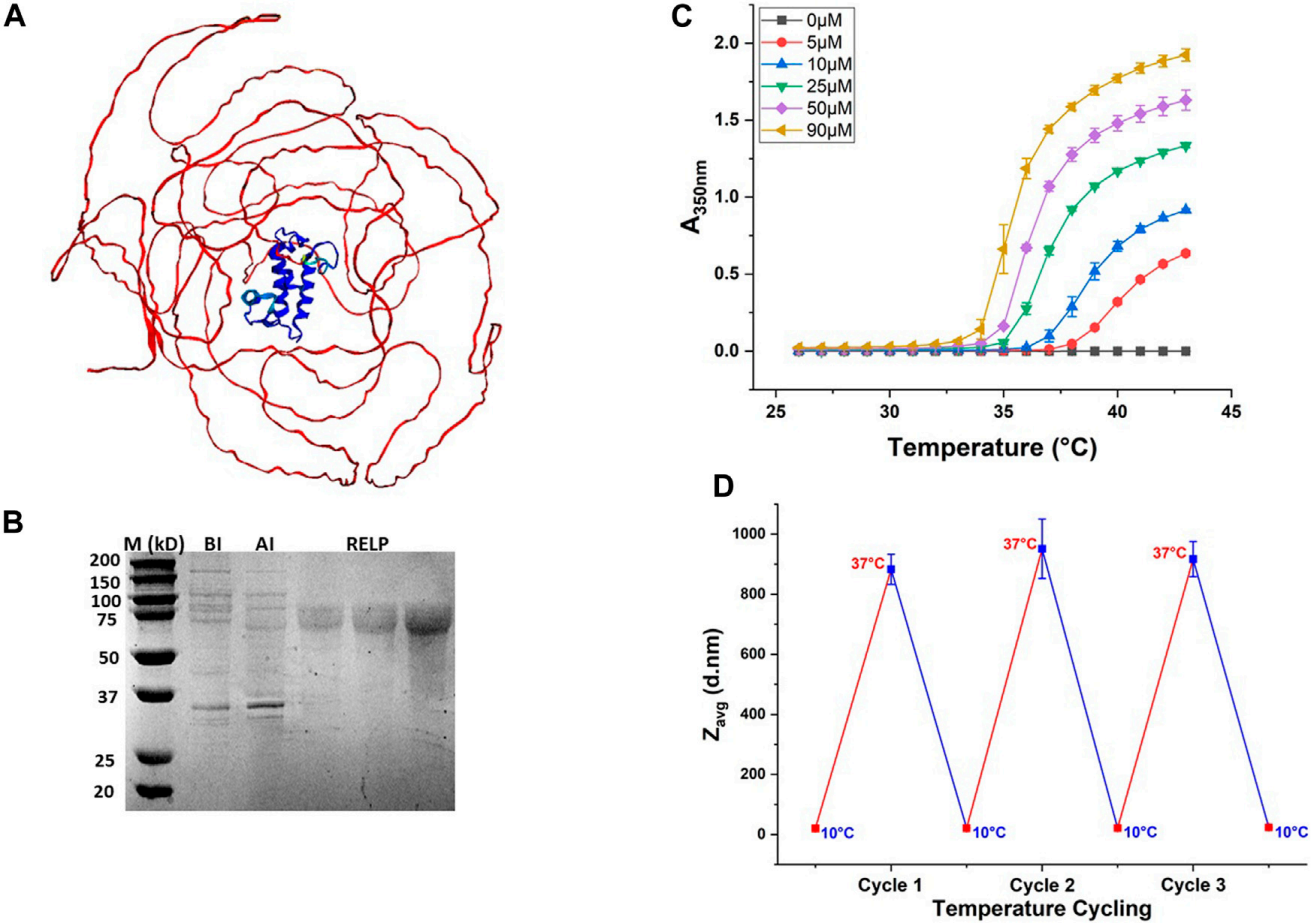
Purification and phase transition behavior of the RELP. **(A)** Predicted 3D structure of the RELP monomer generated using AlphaFold2. **(B)** Purified RELP by SDS-PAGE analysis. From left to right, lane 1: molecular weight marker (M); lane 2: before induction (BI); lane 3: after induction (AI); lane 4–6: purified RELPs obtained from three different batches of purification. **(C)** Turbidity profile for the purified RELP at different concentrations. **(D)** Reversible size change of the RELP over multiple cycles at below Tt (10°C) and above Tt (37°C).

The RELP used in this study is the LanM, derived from *Methylorubrum extorquens* AM1, fused to the C-terminus of the ELP. The generated *de novo* 3D structural model using AlphaFold2 (DeepMind) (Jumper et al., 2021) is shown in Figure 2A. As expected, the predicted structure showed an unstructured ELP region and the folded LanM protein region, suggesting that ELP fusion does not disrupt the folded structure of LanM. The RELP contains the ELP gene encoded with Val as the guest residue and comprises 150 repeats of the [GVGVP] amino acid motif (ELP[V150]). The RELPs purified through ITC have theoretical molecular weights of 75 kDa and were observed in the protein gel around the 75-kDa protein standard, with a purity above 95% (Figure 2B).

As discussed above, the phase transition of an ELP fusion protein is generally modulated by increasing the solution temperature above the inverse T_t_, and by increasing the salt concentration, the transition is achieved even at temperatures much lower than T_t_ (Hassouneh et al., 2010). In the current study, we triggered the coacervation of the RELP by increasing the temperature of the purified protein solution above T_t_, without adding additional salt. For this purpose, following purification, the T_t_ values of the RELP were measured by temperature-programmed turbidimetry. This technique monitors A_350nm_ of the RELP solution while the temperature increases. As T_t_ is concentration-dependent, T_t_ is characterized by a concentration series. The turbidity profile exhibits a sharp increase corresponding to the RELP phase transition from soluble to insoluble aggregates. The results showed that T_t_ of a RELP decreases as its concentration in solution increases (Figure 2C). The reversibility of the RELP phase transition is confirmed by measuring size changes as the ELP transitions from unimer to micron-scale aggregates below T_t_ and above T_t_, respectively. The micrometer-scale coacervates of the RELP were observed at 37°C via DLS analysis. The DLS analysis also showed that the RELP maintains its reversible phase transition property during three cycles of temperature change. An insoluble coacervate is fully reversible upon cooling of the solution (when the solution temperature was decreased below T_t_) and was completely resolubilized (Figure 2D). These results demonstrated that the LLPS of the RELP can be triggered by temperature only, and such a reversible transition of the RELP will improve the existing method for the repeated use of the RELP for selective recovery of REEs.

### 3.2 Reusability of biosorbents for REE recovery from fly ash over multiple cycles

**FIGURE 3:**
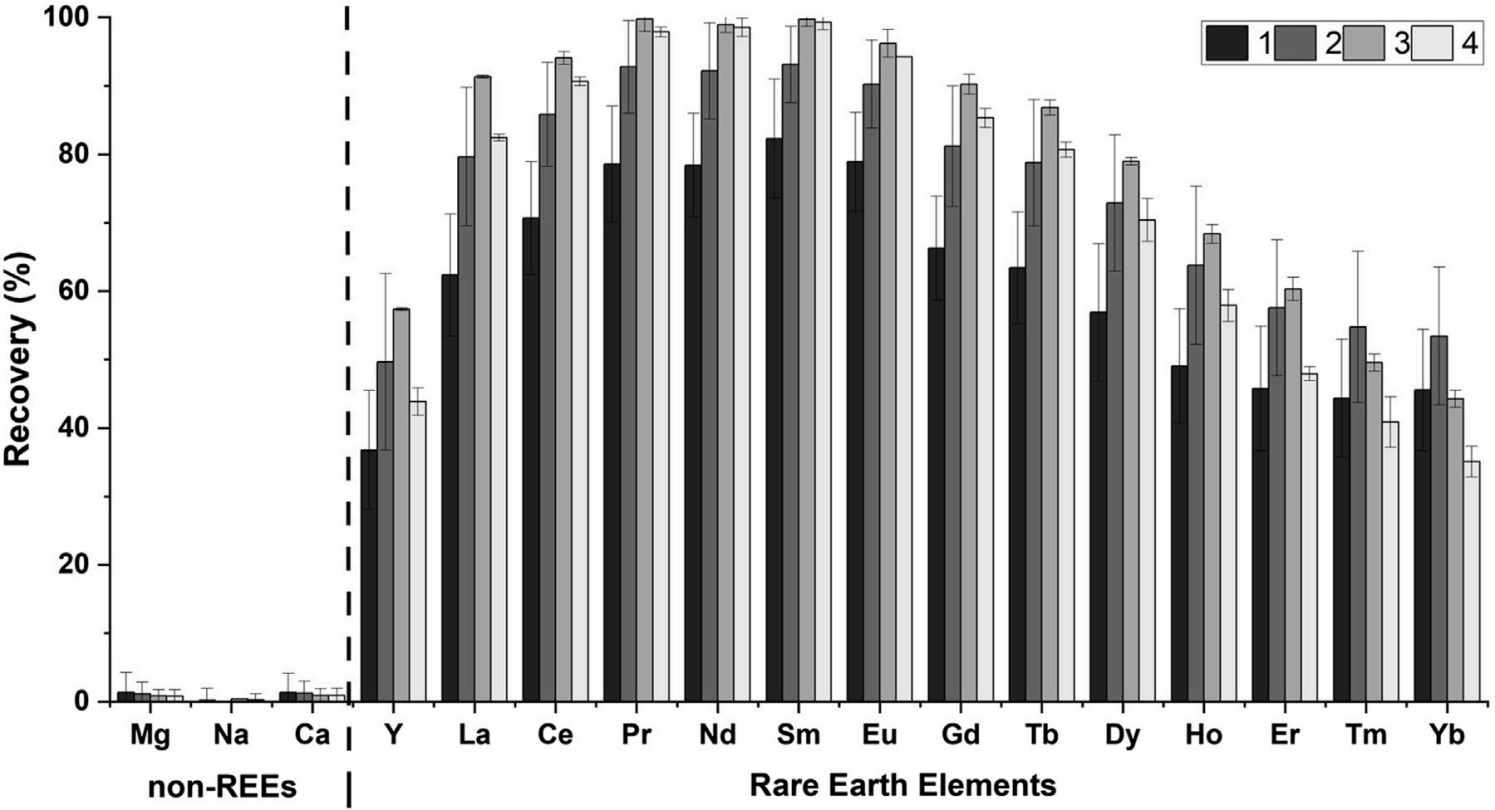
Recovery of REEs by RELP (25 µM) from the coal fly ash leachate (pH = 4.5). The recovery percentage was calculated from the amount of metal recovered relative to the amount added. The data are represented as the mean ± SD and were subjected to one-way ANOVA with Tukey’s *post hoc* test (no significant difference among the four cycles for each REE).

We investigated the REE recovery using the RELP from the coal fly ash leachate, an abundant and potentially valuable industrial REE feedstock. The acid leachate of FA (pH 4.5) in this study contained ≈152 mM of total metal ions (Table 1), including ≈5 µM REEs (0.003 mol% REEs, excluding monovalent ions) and other metals (4 mM Mg, 128 mM Na, and 19 mM Ca). Although the original coal fly ash leachate had Al, Si, and Fe, they were removed during pH adjustment. The RELP retained ∼95% of the initial REE binding capacity even after four cycles (Figure 3). The RELP selectively recovered the REEs for repeated cycles and showed minimal recovery for non-REEs, with no substantial decrease in its performance after four cycles of REE recovery from the FA leachate; the results were insignificant for each element between each cycle (Figure 3). The slight, apparent high recovery efficiency for light REEs (Nd and Sm) over heavy REEs (Figure 3) is likely due to the LanM, derived from *M. extorquens* AM1, favoring the larger and more abundant light REEs than heavy REEs (Gd–Yb) (Table 1), as previously reported (Deblonde et al., 2020; Dong et al., 2021).

In comparison, previous studies showed single-step selective recovery of REEs from complex industrial wastes (Hussain et al., 2022). In a single-step process, the RELP has shown high recovery efficiency for REEs (≈80%) from steel slag leachate containing 41 mM of metal ions (Hussain et al., 2022). Moreover, in another study, the incubation of LanM with leachates of lignite and electronic waste, followed by a single-size exclusion filtration step (using a centrifugal filter with a molecular weight cutoff), showed that all of the non-REE elements remained in the recovered filtrates, and REEs are selectively extracted by the protein fraction left in retentate (Deblonde et al., 2020). The retentates obtained from the filtration assay were digested and used for metal quantification by ICP-MS (Hemmann et al., 2023). Hence, regarding the reusability of biosorbents for the selective and repeated recovery of REEs from complex industrial wastes, this study outperformed comparable REE extraction processes using protein-based technologies (Deblonde et al., 2020; Hussain et al., 2022; Hemmann et al., 2023). Moreover, the method we used is entirely protein-based, and hence, it does not require any centrifugal filter to separate protein-bound REEs from unbound non-REEs or proteins from recovered REEs after the desorption step. Considering industrial applications, the simplistic non-chromatographic purification and high stability upon reuse of the RELP demonstrate the flexibility and potential for use as a low-cost recovery platform for REEs from complex waste sources.

### 3.3 Biosorbent for the recovery of high-purity REEs in extremely low-concentration samples

REEs have been reported to exist at very low concentrations (picomolar scales) in various low-grade REE sources. We investigated the ability of the RELP to recover REEs from environmentally relevant concentrations (µM to nM range). The RELP was incubated in different solutions containing varying concentrations of REEs (Tb^3+^) (µM to nM range) and equimolar concentrations of Mg and Zn (∼1 mM each) at pH 5.8. Mg and Zn were selected as representative competing non-REE elements because REEs frequently coexist with these metals at high concentrations (high μM range) in ore and waste streams. The RELP selectively recovers the REE (Tb^3+^), which has a concentration as low as 1.8 nM (Table 2). Mattocks et al. constructed a LanM-based protein sensor. They confirmed its detection efficiency for REEs using fluorescence resonance energy transfer. The sensor detected REEs within a 10–50 µM range, with a 7-fold ratiometric response but only a weak response to divalent and trivalent metal ions at pH 7.0 (Mattocks et al., 2019). Moreover, in another study, Trp-substituted LanM directly quantified 3 ppb (18 nM) terbium in acid mine drainage using luminescence resonance energy transfer at pH 5.0 (Featherston et al., 2021). These studies anticipate that LanM can be used for detecting and quantifying REEs in extremely low concentrations in environmental and industrial samples. The results suggest the advantage of the RELP system for the selective recovery of high-purity REEs from extremely low-concentration metal solutions. We anticipate further applications of this system by re-engineering the RELP to selectively recover other metal ions (e.g., actinides) present at extremely low levels in seawater (Mattocks et al., 2022). Most previous biosorbent studies are more focused on environmental remediation than the extraction of REEs for industrial purposes, where the purity of the recovered metals is critical. The lanthanide-binding tag-engineered *Escherichia coli* cells showed REE purity of 0.11% in the extracted solution because of the adsorption of non-REEs to the cell surface, which eluted along with REEs (Brewer et al., 2019b). In terms of purity, LanM-immobilized magnetic nanoparticles showed 10.9 mol% REE purity in the recovered solution, which was 1,155-fold higher than in the FA leachate stock solution (0.009 mol%) (Ye et al., 2023a). The LanM-immobilized agarose microbead column showed 88.2 mol% REE purity, which was 2,040-fold higher than in the FA leachate stock solution (0.043 mol%) (Dong et al., 2021). The increase in REE purity by the RELP was 10–100 orders of magnitude higher than other benchmark materials, such as LBT-engineered cells and LanM-immobilized magnetic nanoparticles, and comparable to the LanM-based column. Notably, the fold increase in REE purity was also 10–100 order-of-magnitude higher than that of LanM-based biosorbents (Table 2). The results demonstrated that the RELP can be used to obtain high-purity REE mixture solutions by utilizing low-grade REE solutions. However, the RELP-based approach has limitations, such as the inability for intra-REE separation, as was demonstrated by the LanM-immobilized resin. We anticipate achieving even higher yields and product purity for other environmental and industrial waste leachates, which will be the subject of future studies.

**TABLE 2:** REE (Tb^3+^) purity after recovery by the RELP. The initial molar concentrations of Tb^3+^ in each batch of synthetic solutions (ppb) and the amount of Mg^2+^ and Zn^2+^ added to each solution are approximately 1 mM. The experiments were conducted in triplicates, and values are represented as the mean.

Initial Tb^3+^ concentration µM (ppb)	Tb^3+^ purity in initial solution (%)	Tb^3+^ purity in recovered solution (%)	Fold increase in Tb^3+^ purity	Recovery of Tb^3+^ (%)
0.94 ± 1.9*10^−2^ (150)	0.044 ± 1.6*10^−3^	1.0*10^2^	2250 ± 82	78 ± 1.59
9.4*10^−2^ ± 1.9*10^−3^ (15)	4.0*10^−3^ ± 1.6*10^−4^	1.0*10^2^	22491 ± 828	77 ± 1.56
1.8*10^−2^ ± 3.8*10^−4^ (3.0)	8.9*10^−4^ ± 3.2*10^−5^	1.0*10^2^	112452 ± 4141	63 ± 1.28
1.8*10^−3^ ± 8.6*10^−5^ (0.29)	8.8*10^−5^ ± 3.9*10^−6^	2.1*10^−3^ ± 1.5*10^−5^	23 ± 0.99	71 ± 3.44

It is noteworthy that the RELP platform can be leveraged to develop a more efficient biosorbent for recovering other critical elements and isotopes, provided it is re-engineered with other lanmodulin variants (Mattocks et al., 2022; Park et al., 2022).

## 4 Conclusion and environmental implications

In this study, we demonstrated the RELP as a promising technology for the highly selective extraction of REEs from low-grade REE feedstocks. Current hydrometallurgical and electrochemical technologies are environmentally destructive, have poor selectivity for REEs, and are inefficient for dilute and low-grade feedstocks. Compared to existing LanM-based biosorption approaches, this process requires fewer chemical and energy inputs and generates minimal byproducts or waste solutions. The RELP also demonstrated distinct advantages, including upscale non-chromatographic protein purification and reuse and ease of separation. Notably, the RELP effectively and selectively extracts REEs from the low-grade FA leachate over multiple cycles. The RELP shows potential for REE recovery from other low-grade waste streams (leachate concentrations of REEs <1%) and containing high non-REE levels. In particular, the RELP biosorbent could serve as a platform for extracting high-purity REEs, such as dilute and low-grade REE solutions, and transforming them into more highly pure REE solutions. The recovered REE solution can be precipitated as mineral phases by adding oxalate or carbonate, and subsequently, the precipitates can be roasted to obtain total rare earth oxides. The simplicity, scalability, and economic viability of the method make it promising for industrial adoption. Purification and high reusability are required properties for developing low-cost biosorption technology for REE separation. However, properly designing fermenters with temperature-controlled purification and recovery of REEs will be another scaling-up strategy. This research aligns with UN Sustainable Development Goal 12, promotes a circular economy for a sustainable future, and reflects the role of green chemistry and engineering in sustainable production. By enabling the extraction of critical elements from diverse sources, RELP technology contributes to the sustainable management of REE-containing waste and diversifies the REE supply chain by utilizing fly ash as REE feedstock.

## Data Availability

The original contributions presented in the study are included in the article/Supplementary Material; further inquiries can be directed to the corresponding author.
