# Supplementary material for: Recovery of rare earth elements from low-grade coal fly ash using a recyclable protein biosorbent
Source: Front Bioeng Biotechnol. 2024 May 16;12:1385845. doi: 10.3389/fbioe.2024.1385845 (PMC11137179; doi:10.3389/fbioe.2024.1385845)
Supplement: Supplementary file 1 [file DataSheet1.docx]

Supplementary Material

**Recovery of Rare-Earth Elements from Low-Grade Coal Fly Ash Using a Recyclable Protein Biosorbent**

Zohaib Hussain^1^, Divya Dwivedi^1^, Inchan Kwon^1*^

^1^School of Materials Science and Engineering, Gwangju Institute of Science and Technology (GIST), 123 Cheomdangwagi-ro, Buk-gu, Gwangju 61005, Republic of Korea

*Correspondence:

**Inchan Kwon**

Professor

School of Materials Science and Engineering

Gwangju Institute of Science and Technology (GIST)

Gwangju 61005, Republic of Korea

Phone: +82 62-715-2312

Fax: +82 62-715-2304

Email: [inchan@gist.ac.kr](mailto:inchan@gist.ac.kr)

#
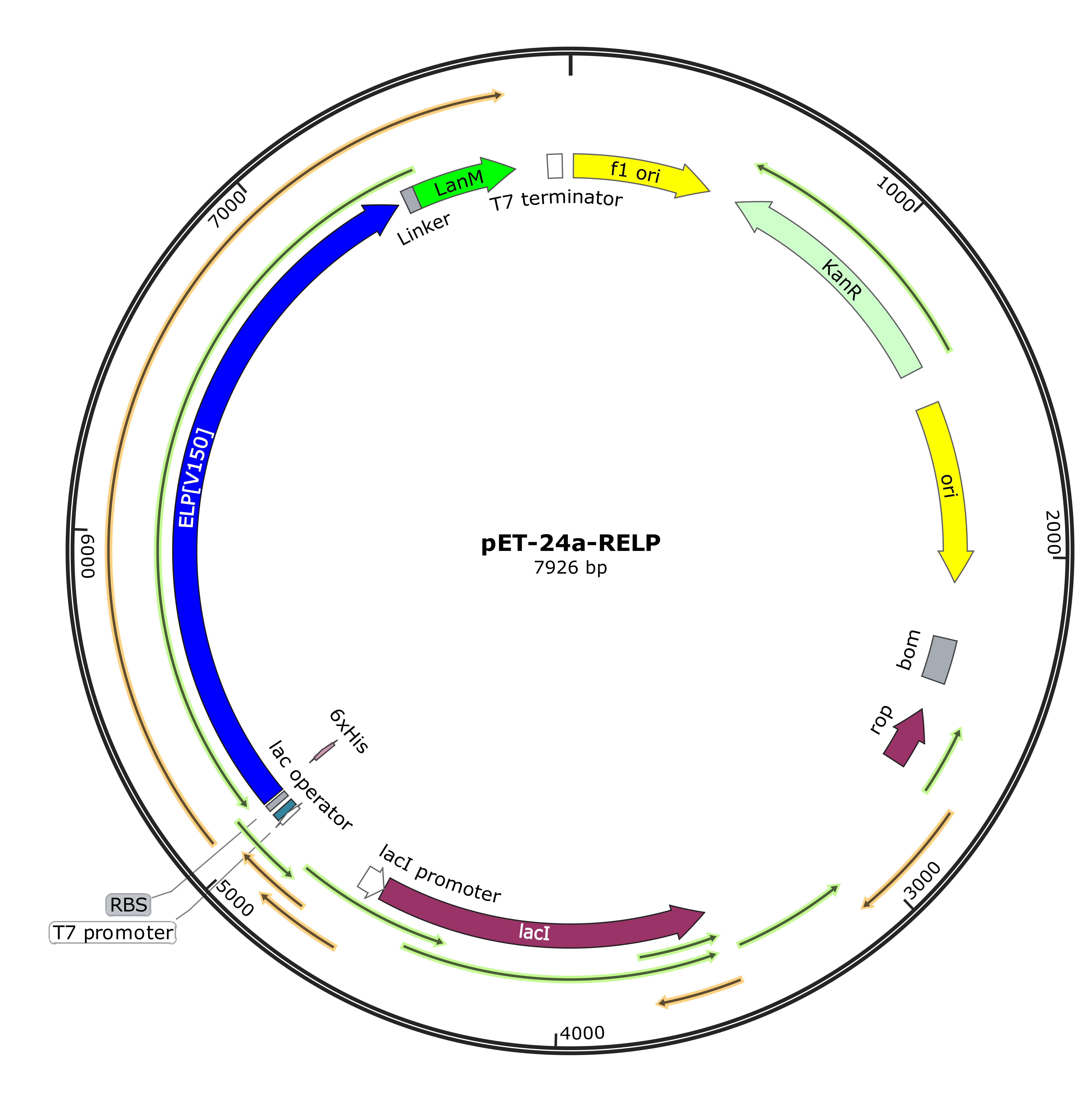


**Name**  **Gene Product**

Lac operator bound moiety lac repressor encoded by lacI

RBS ribosome binding site from bacteriophage T7 gene

ELP[V150]

Linker

Lanmodulin LanM binding protein

Tag 6xHis 6xHis affinity tag

Terminator T7 transcription terminator for bacteriophage T7 RNA polymerase

Antibiotic resistance KanR aminoglycoside phosphotransferase

ori origin of replication

bom basis of mobility region from pBR322

rop rop protein maintains plasmids at low copy number

LacI promotor LacI

LacI LacI LacI repressor

**Supplementary Figure 1.** pET-24a-RELP plasmid.
